# Supplementary material for: Causal interplay between lactose intolerance and gut microbiota: a combined bidirectional Mendelian randomization and in vivo validation study
Source: Front Nutr. 2026 Jun 1;13:1803337. doi: 10.3389/fnut.2026.1803337 (PMC13265576; doi:10.3389/fnut.2026.1803337)
Supplement: Supplementary file 1 [file Data_Sheet_1.zip › supplementary materials/Forward/forest plot/ebi-a-GCST90027670.finngen_R12_E4_LACTONAS.pdf]

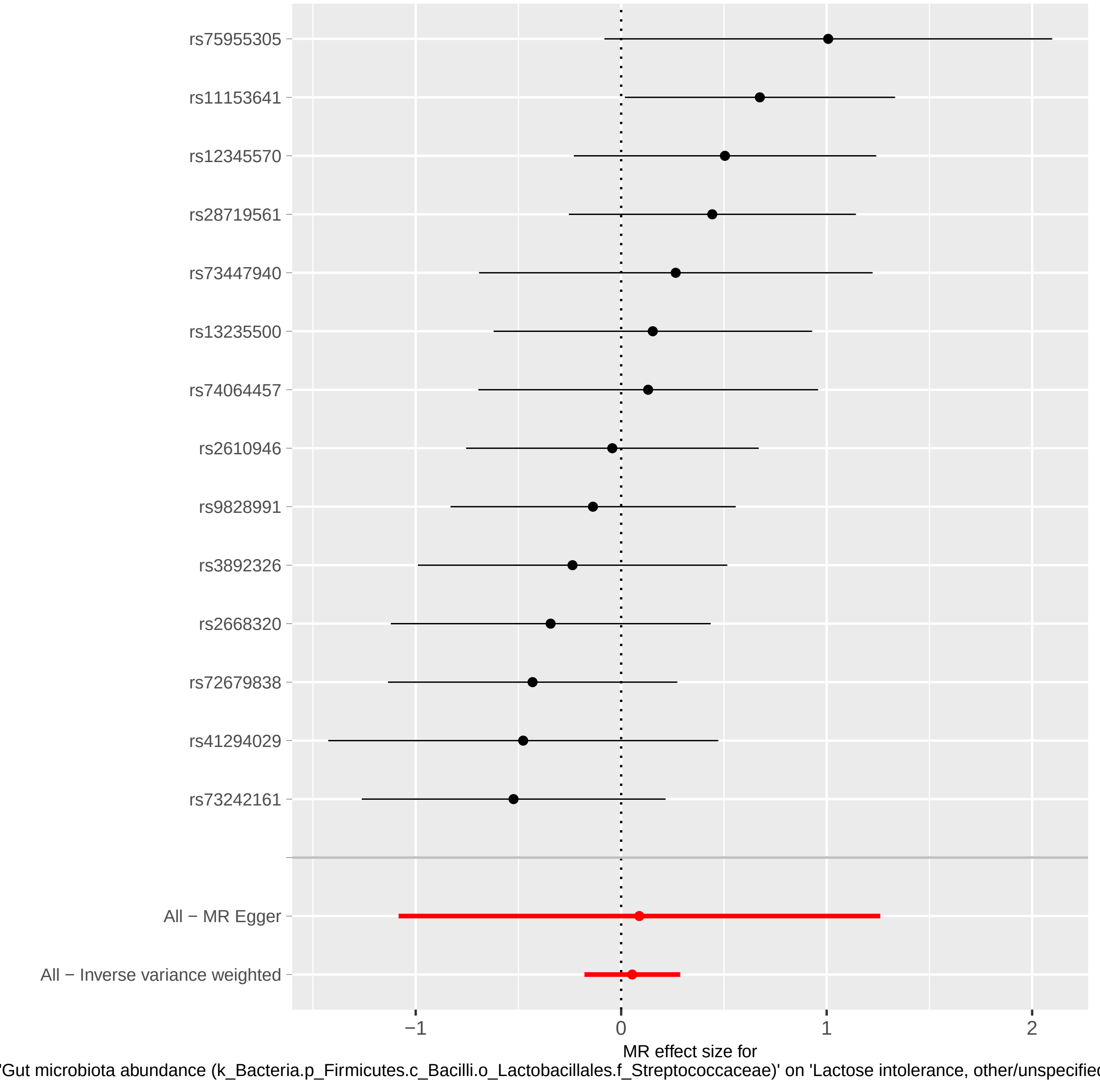

rs75955305
rs11153641
rs12345570
rs28719561
rs73447940
rs13235500
rs74064457
rs2610946
rs9828991
rs3892326
rs2668320
rs72679838
rs41294029
rs73242161
All − MR Egger
All − Inverse variance weighted
−1
0
1
2
MR effect size for
'Gut microbiota abundance (k_Bacteria.p_Firmicutes.c_Bacilli.o_Lactobacillales.f_Streptococcaceae)' on 'Lactose intolerance, other/unspecified
